# Enhanced Skin Permeation of Anti-wrinkle Peptides via Molecular Modification

**DOI:** 10.1038/s41598-017-18454-z

**Published:** 2018-01-25

**Authors:** Seng Han Lim, Yuanyuan Sun, Thulasi Thiruvallur Madanagopal, Vinicius Rosa, Lifeng Kang

**Affiliations:** 1 Department of Pharmacy, Faculty of Science, National University of Singapore, Singapore, 117543 Singapore; 2 Department of Pharmacy, Tongji Hospital, Tongji Medical College, Huazhong University of Science and Technology, Wuhan, 430030 China; 3 Faculty of Dentistry, National University of Singapore, Singapore, 119083 Singapore; 4 Present Address: Faculty of Pharmacy, University of Sydney, Pharmacy and Bank Building A15, Sydney, NSW 2006 Australia

## Abstract

Wrinkles can have a negative effect on quality of life and Botox is one of the most effective and common treatments. Argireline (Arg0), a mimetic of Botox, has been found to be safer than Botox and effective in reducing wrinkles, with efficacies up to 48% upon 4 weeks of twice daily treatment. However, the skin permeation of Arg0 is poor, due to its large molecular weight and hydrophilicity. Arg0 exists in zwitterionic form and this charged state hindered its skin permeation. Chemical modification of the peptide structure to reduce the formation of zwitterions may result in increased skin permeability. We investigated a total of 4 peptide analogues (Arg0, Arg1, Arg2, Arg3), in terms of skin permeation and wrinkle reduction. The 4 peptides were dissolved in various propylene glycol and water co-solvents. Enhanced human skin permeation was demonstrated by both Arg2 and Arg3 *in vitro*. On the other hand, the abilities of the 4 analogues to reduce wrinkle formation were also compared using primary human dental pulp stem cells derived neurons. By measuring the inhibition of glutamate release from the neurons *in vitro*, it was shown that Arg3 was the most effective, followed by Arg1, Arg0 and Arg2.

## Introduction

Wrinkles are visible creases or folds in the skin^1^ and they are often the first sign of ageing^2^. Changes in physical appearance due to wrinkles can have a negative effect on the quality of life. In some cases, concerns over physical appearances can affect personal interactions, occupational functioning and self-esteem^3^. By far, Botox has been one of the most effective and commonly administered compounds to reduce wrinkles in the United States, with up to 2.6 million injections in 2011^4^. However, Botox is often questioned for its safety in human, due to its high toxicity and therefore, its use must be under strict control^5^.

Argireline^®^ (Arg0), developed as a topical mimetic of Botox, is a synthetic acetyl hexapeptide, patterned after the N-terminal end of the protein Synaptosomal-associated protein 25 (SNAP-25). Arg0 competes with SNAP-25 for the binding with vesicle-associated membrane protein (VAMP). This destabilises the formation of a ternary Soluble N-ethylmaleimide-sensitive factor Attachment protein REceptor (SNARE) complex resulting in the inhibition of neuronal exocytosis. As a result, Arg0 inhibits the release of acetylcholine^6^ and reduces repetitive contraction of the intrinsic muscles of facial expression, thereby decreases hyperkinetic facial lines or expression wrinkles^7^. However, unlike Botox, the acute toxicity is insignificant (≥2000 mgkg^−1^ for Arg0 versus 20 ngkg^−1^ for Botox)^6^. In addition, Arg0 has been found to be effective in reducing wrinkles, with efficacies up to 48% upon 4 weeks of twice daily treatment^6,8–10^.

While Arg0 has good efficacy and safety profiles, the permeation of Arg0 through skin is poor. With a high molecular weight of 889 Dalton and a low LogP value of −6.3, Arg0 remains mainly on the surface of the skin upon topical application^11^. In an *in vitro* skin permeation study using human cadaver skin samples, it was found that the majority of Arg0 remained on the surface of the skin and was washed away subsequently. Only 0.22% of the total amount permeated through the skin and retained within stratum corneum, the topmost layer skin; 0.01% of the peptide made it through to the epidermis^12^. The poor skin permeation can lead to a significant wastage of peptide, resulting in unwanted high cost of production and an ineffective final dosage form.

To overcome the problem, several research groups have attempted to enhance transdermal delivery of Arg0 by optimising the formulations. Hoppel *et al*. showed a clear superiority of water-rich water-in-oil-in-water (W/O/W) and oil-in-water (O/W) emulsions over oil-rich water-in-oil emulsion (W/O) emulsions, due to increased absorption of water-rich emulsions into the skin^13^. However, there was no comparison to the pure Arg0 solution and no comparison could be drawn to see the effect of emulsion. Kraeling *et al*. used a high concentration of Arg0^12^ (at 10% w/w in an O/W emulsion), yet, the results were disappointing when only 0.23% of the total amount of Arg0 permeated through and stayed in the human epidermis. Also, this approach may result in costly products. Ruiz *et al*. attempted to incorporate Arg0 into either a cream or gel dosage form and demonstrated that a cream based dosage form is superior to a gel dosage form in transdermal delivery of Arg0^14^. While these methods were relatively simple to perform, the increase of Arg0 delivery through skin was unsatisfactory.

Other than modifying the formulation, several techniques have been put forth to overcome the stratum corneum for other peptide drugs, for e.g., chemical enhancers, iontophoresis^15^, microneedles^16–19^, sonophoresis^20^, thermal ablation^21^, radiofrequency ablation^22^, jet injectors^23^ and electroporation^24,25^. A number of commercial iontophoretic devices have been available for transdermal delivery, although their market penetration has been limited because of cost and technical issues^15^. Other physical methods, such as electroporation or ablation, are generally inconvenient to users as they require special machineries and expertise to perform. In addition, microneedle is a physical enhancement method which creates micro-pores in the skin in a minimally invasive manner to deliver peptides. However, the idea of placing needles on the face may be of concern to users.

Another approach to enhance transdermal delivery of Arg0 is structural modification of the amino acid side chains. Arg0 exists in zwitterionic form and this charged state makes it very hydrophilic and poses a major obstacle for transdermal delivery, with the other being its high molecular weight. Chemical modification of the peptide structure to reduce the formation of zwitterions and/or to increase lipophilicity may result in increased permeability of Arg0 into the skin.

**Figure 1:**
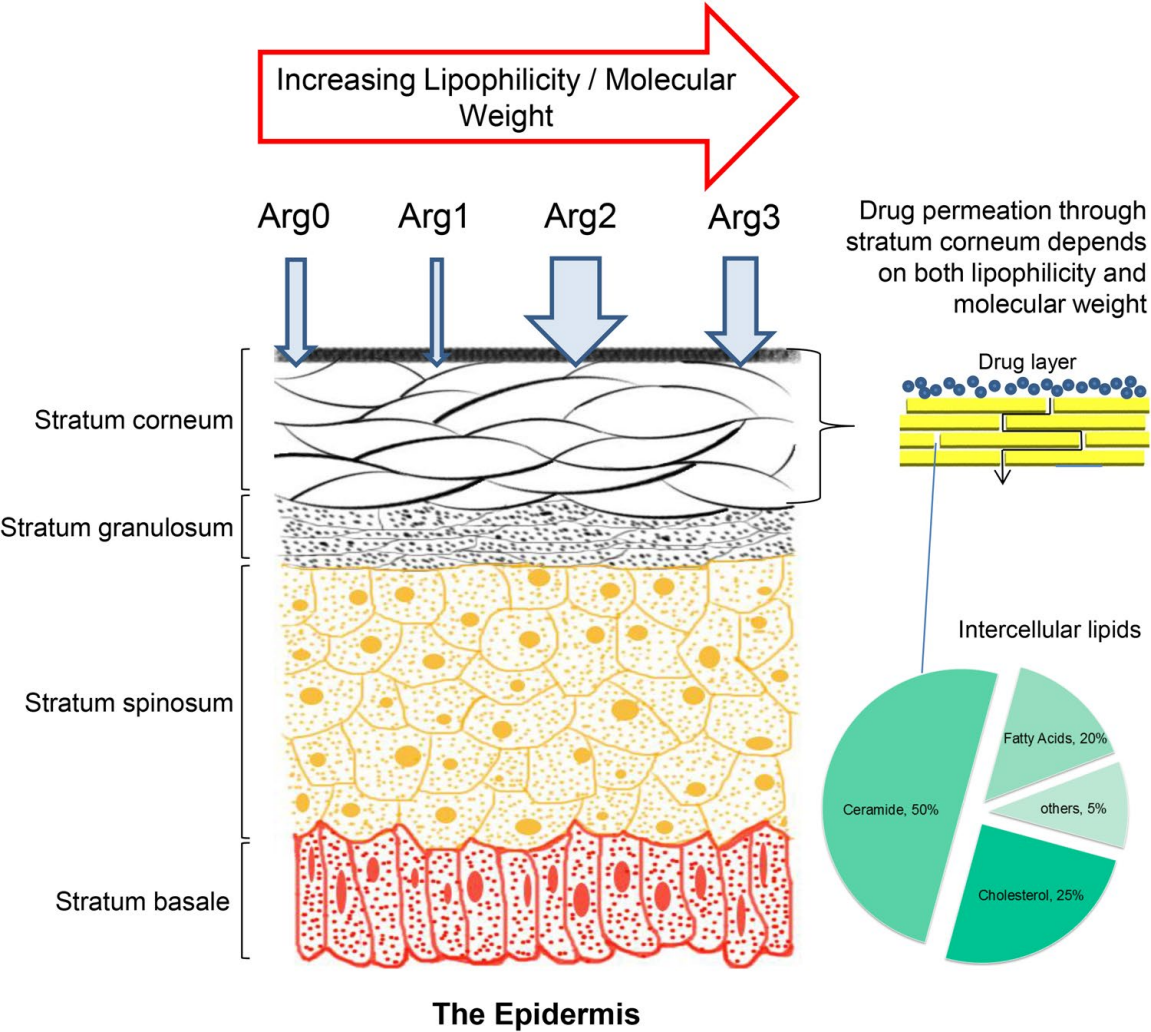
Overall schematic for the enhancement of transdermal delivery of small peptide through chemical structure modification.

In this study, we investigated a total of 4 peptide analogues (Arg0, Arg1, Arg2, Arg3), on their abilities to permeate through skin and their *in vitro* efficacies on neuronal cell cultures. Each of these analogues has different amino side chain functional groups, with increasing lipophilicity and molecular weight (Fig. 1). The 4 peptide analogues were dissolved in various propylene glycol (PG) and water (H_2_O) co-solvent systems, as PG is widely used as co-solvent and permeation enhancer^26^ in the cosmetics. Further, PG is generally regarded as safe and does not cause irritation or side effects even at high concentrations^27^.

## Results

### Structural Modification of Amino Acid Side Chains

**Figure 2:**
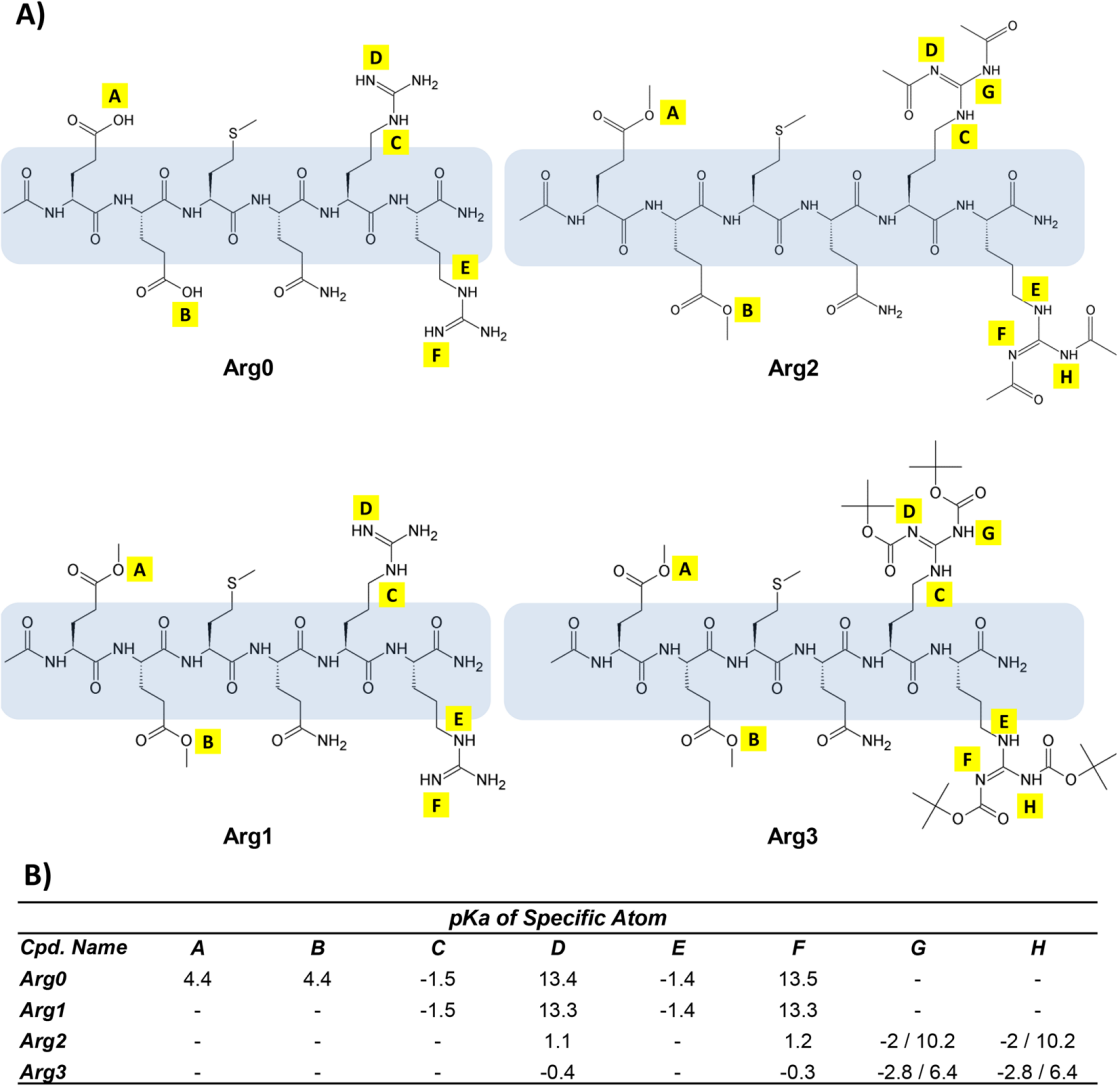
The 4 synthesised peptide analogues. (**A**) Chemical structures of parent compound (Arg0) and its chemical analogues. For all 3 compounds, Arg1, Arg2, Arg3, 2 esters functional groups have been added to the chemical structure to replace the 2 carboxylic acids at position A&B. (**B**) A table summary of the purity of synthesised peptide analogues and the various pKa changes to the specific atoms of the amino acid side chains. In general, the pKa favours that of deprotonation, thus reducing the formation of charged ions. *In silico* prediction of pKa is performed via ACD/Labs Percepta platform.

Figure 2A shows the chemical structures of the 4 peptide analogues, Arg0, Arg1, Arg2 and Arg3. The 3 peptide analogues were modified from their parent compound, namely, Arg0, to reduce the formation of zwitterions and increase the lipophilicity. In all 3 analogues (Arg1, Arg2, Arg3), the carboxylic acid functional groups (marked as position A & B, Fig. 2A) were esterified. In addition to esterification, Arg2 and Arg3 had 2 more structural modifications each. In Arg2, the guanidine functional group was modified to become an acetamide functional group for each end of the guanidine’s end nitrogen. In Arg3, the guanidine functional group was modified into a bis-carbamate functional group ending with a tertiary butyl group for each carbamate functional group.

### Physicochemical Properties of Peptide Analogues

A substantial decrease in pKa of ionisable atoms in the amino acid side chains was observed after structural modification (Fig. 2B). After esterification of the side chain, as seen in Arg1, Arg2 and Arg3, the originally ionisable atom A & B no longer form charged ions. In addition, after a second structural modification as seen in Arg2 and Arg3, comparing atom D & F, the pKa dropped from ~13 to ~0.5. A smaller pKa implied that the atom will less likely protonate and therefore less likely form charged ions. For atoms G & H for both Arg2 and Arg3, they have very low pKa and hence, were also unlikely to protonate to form charged ions. Table 1 shows the basic physicochemical properties of the 4 peptide analogues. From Arg0 to Arg3, there was an increasing trend of lipophilicity and molecular weight. Arg3 had the most optimal LogP of 1.75, while both Arg1 and Arg2 had LogP values less hydrophilic than that of Arg0 (−4.67 and −4.18 VS −6.37). However, all 4 analogues were high in molecular weight due to the nature of the peptide, with Arg3 being the highest (MW: 1317.51), followed by Arg2 (MW: 1085.19), Arg1 (MW: 917.05) and Arg0 (888.99).

**Table 1:** Physicochemical properties of the 4 chemical analogues, based on *in silico* prediction by ACD/Labs Percepta platform. In general, all 4 analogues do not have significant potential for skin/eye irritation and mutagenecity, except for Arg1. Arg3 has the most optimal LogP but also with the highest molecular weight.

Physicochemical Properties of Various Chemical Analogues				
Properties	Arg0	Arg1	Arg2	Arg3
LogP	−6.37	−4.67	−4.18	1.75
MW	888.99	917.05	1085.19	1317.51
H-donors	20	18	14	14
H-acceptors	26	26	30	34
Rot. Bonds	34	36	40	48
Rings	0	0	0	0
Solubility	57.9 mg/mL	1000 mg/mL	0.008 mg/mL	0.0002 mg/mL
AMES	0.27	0.40	0.38	0.37
Skin irritation	0.09	0.52	0.13	0.09
Eye irritation	0.01	0.06	0.01	0.00

### *In Vitro* Skin Permeation of Peptide Analogues

**Figure 3:**
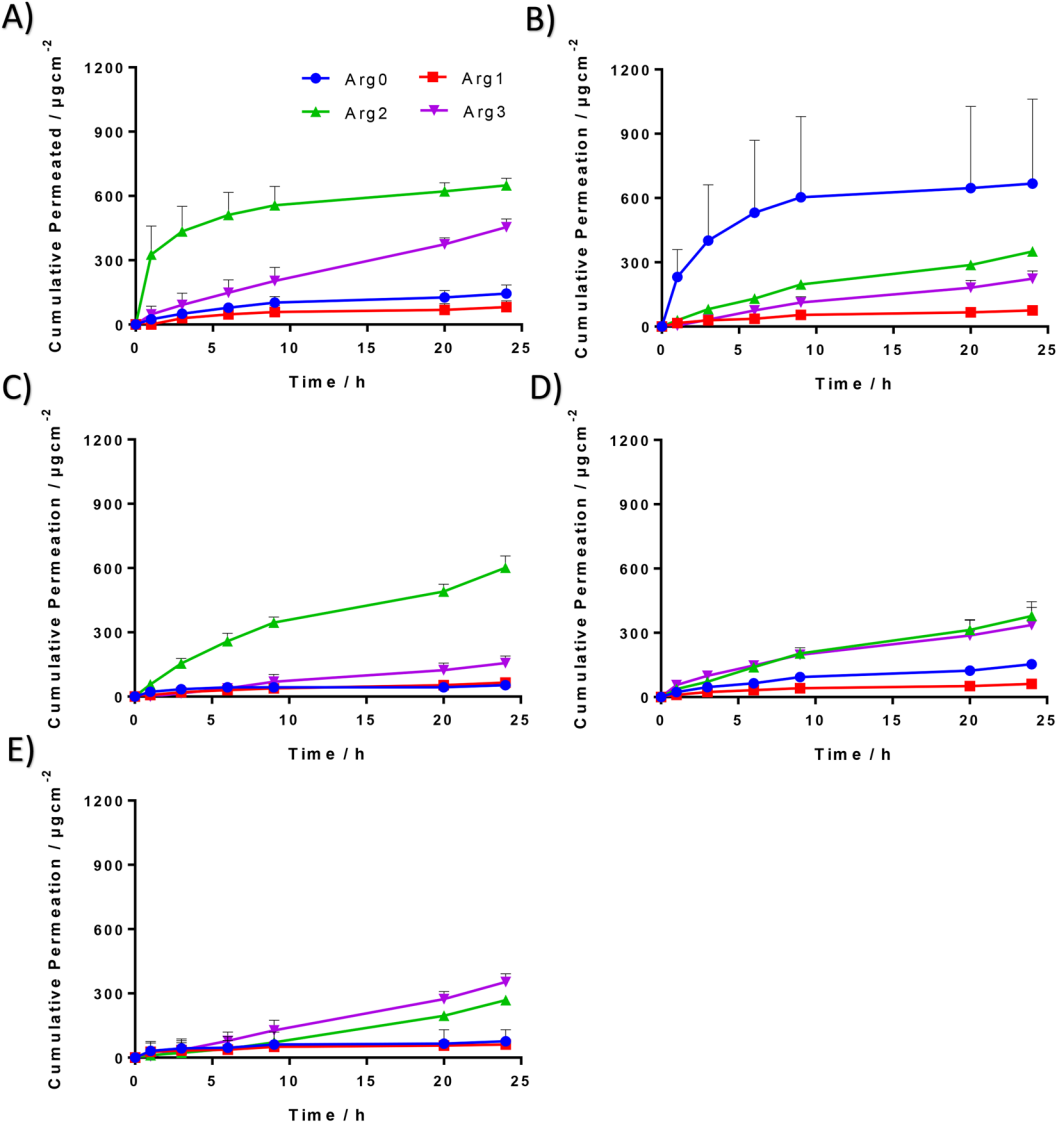
Cumulative release profiles of the 4 different peptide analogues over a time period of 24 hrs, in varying concentrations of PG:H_2_O co-solvent systems. (**A**) 100%PG, (**B**) 70%PG, (**C**) 50% PG, (**D**) 30%PG, (**E**) 0%PG. Each coloured curve represents a different cumulative release profile of a different chemical analogue, at the specified propylene glycol concentration.

**Figure 4:**
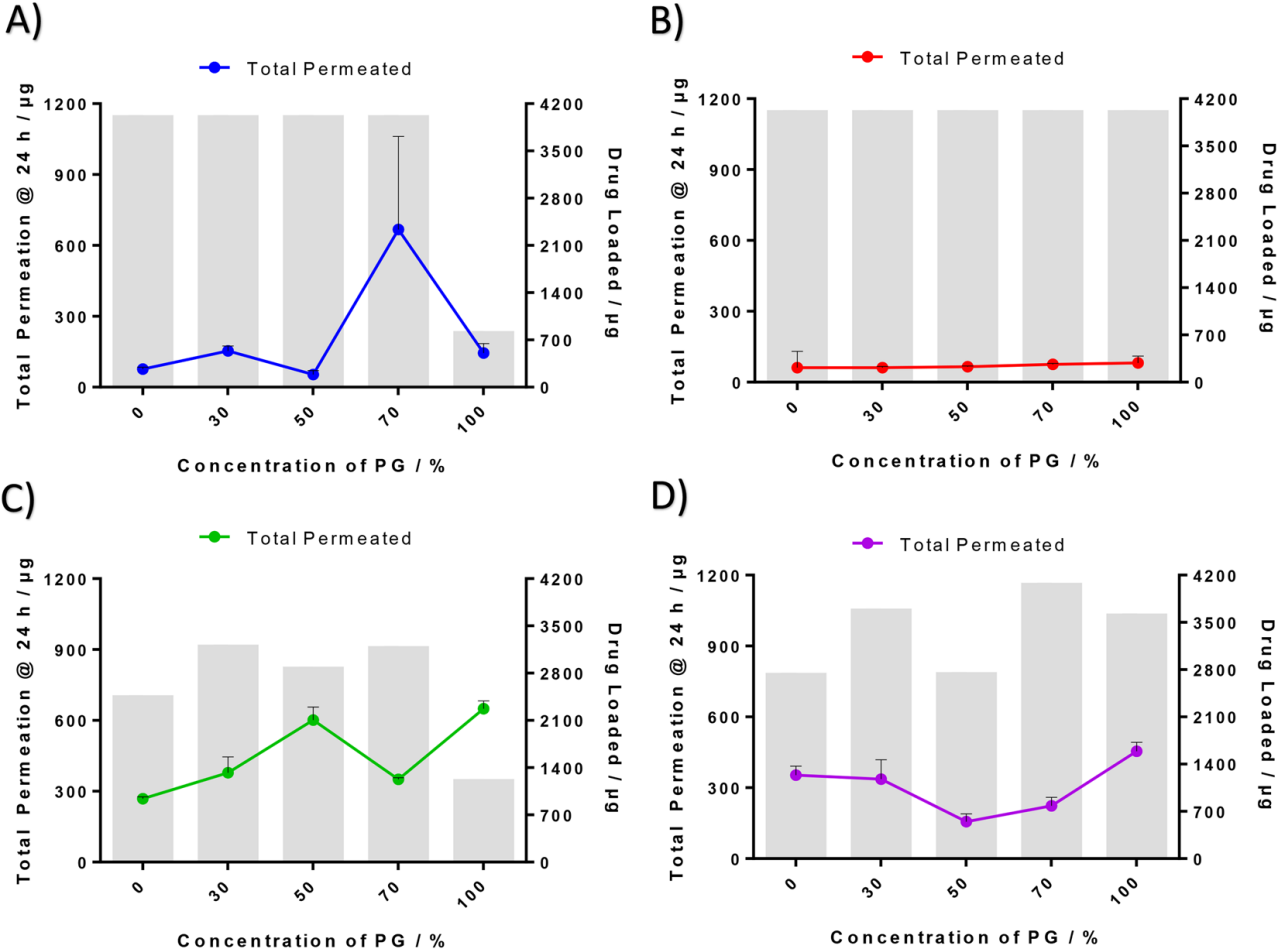
Effects of propylene glycol concentration on the cumulative permeation in 24 hrs and amount of drug in donor cell for different peptide analogues. Line graph: Amount of peptide analogue permeated through the skin after 24 hrs. Bar graph: Amount of drug loaded for each analogue at varying propylene glycol concentrations.

PG is commonly used in cosmetics and is well known for its co-solvent and permeation enhancing effect. Hence, pure PG was also used to prepare the donor peptide solution for each of the 4 analogues (Fig. 3A, Table 2). In pure PG, comparing to Arg0, a significantly higher amount of peptide permeated through skin by the end of 24 hrs (p < 0.01) was demonstrated by both Arg2 (~4.5 times) and Arg3 (~3.1 times). A lower amount of peptide permeated through the skin was observed in Arg1 as compared to Arg0, however, it was not significantly different. The transdermal delivery of all 4 peptide analogues was generally enhanced with the use of pure PG, compared to when the same analogue was dissolved in pure water. Significant difference was observed only for Arg2 (p < 0.01) (Fig. 4C).

**Table 2:** A summary of the cumulative amount of chemical analogues, permeated through the skin after 24 hrs. Statistical comparison (one-way ANOVA, with *post-hoc* Tukey analysis) of the cumulative amount were compared between different chemical analogues within the same concentration of PG:H_2_O co-solvent system. In general, both Arg2 and Arg3 have greater permeation at 24 hrs as compared to Arg0, except in 70% PG co-solvent system where Arg0 performed significantly better than the other chemical analogues.

Cumulative Amount Permeated Through Skin after 24 hrs				
Concentration of PG (%)	Compound No.	Average (µg)	SD	P-value (Compared to Arg0)
0	Arg0	76.4	7.6	1
	Arg1	61.9	69.0	0.969
	Arg2	268.5	8.1	**< 0.01
	Arg3	353.6	38.3	**< 0.01
30	Arg0	153.2	21.4	1
	Arg1	61.6	4.5	0.239
	Arg2	378.2	67.6	**< 0.01
	Arg3	337.0	81.6	* 0.013
50	Arg0	53.5	16.6	1
	Arg1	65.1	3.9	0.972
	Arg2	601.7	54.4	**< 0.01
	Arg3	156.1	33.7	* 0.022
70	Arg0	667.4	393.9	1
	Arg1	75.5	4.5	0.026
	Arg2	350.8	6.9	0.277
	Arg3	223.5	35.5	0.095
100	Arg0	144.4	40.5	1
	Arg1	81.6	29.0	0.218
	Arg2	649.1	33.8	**< 0.01
	Arg3	454.1	38.4	**< 0.01

*p < 0.05.

**p < 0.01.

Varying concentrations of PG in water were also used as solvent to prepare the donor solution for different peptide analogues (Fig. 3B–D, Table 2). A total of 3 other concentrations of PG in water were 30%, 50% and 70%. In 30% PG, a higher cumulative amounts of peptide that permeated through the skin after 24 hrs, as compared to Arg0, were demonstrated by both Arg2 (p < 0.01, ~2.5 times) and Arg3 (p = 0.013, ~2.2 times). On the other hand, compared to Arg0, less than half the cumulative amount of peptide that permeated through the skin after 24 hrs was observed in the Arg1. In 50% PG, significantly higher cumulative amount of peptide that permeated through the skin after 24 hrs, as compared to Arg0, was demonstrated in Arg2 (p < 0.01, 11.2 times) and Arg3 (p = 0.022, ~2.9 times). In 70% PG, the highest cumulative amount of peptide permeated through the skin after 24 hrs amongst the 4 peptide analogues was observed in Arg0. Significantly less cumulative amount of peptide that permeated through the skin by 24 hrs, as compared to Arg0, was observed in Arg1 (p = 0.026). Across all 5 concentrations of PG investigated, a lowest cumulative amount of peptide that permeated through the skin by 24 hrs was consistently observed in Arg1. On the other hand, across all 5 concentrations of PG, higher cumulative amount of peptide that permeated through the skin after 24 hrs, as compared to Arg0, was consistently observed in Arg2 (p < 0.01) and Arg3), with the exception of 70% PG. The effect of PG in enhancing skin permeation of these peptide analogues is not uniform throughout the PG concentrations and the trend varies amongst the 4 peptide analogues. For Arg0, the highest amount of peptide that permeated through the skin at 24 hrs was demonstrated by 70% PG. For Arg1, Arg2 and Arg3 that concentration was 100% PG (Fig. 4).

### *In Vitro* Inhibition of Glutamate Release

**Figure 5:**
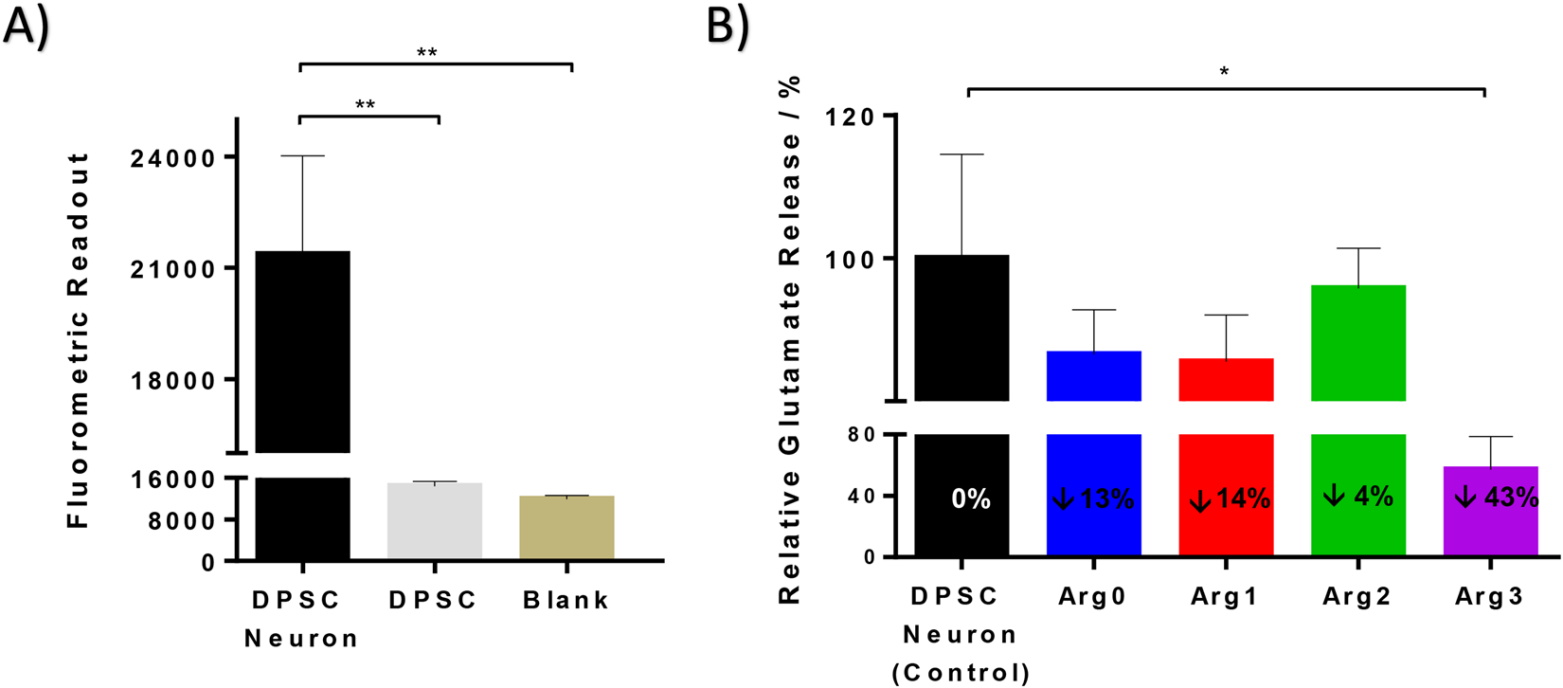
*In vitro* inhibition of glutamate release using dental pulp stem cell derived neurons. (**A**) Comparison of glutamate release between DPSC and DPSC neurons after 12 weeks of differentiation. A significantly higher fluorometric readout (indicative of the amount of glutamate released) was observed in the neuronally differentiated DPSC neuron group, as compared to blank and DPSC group. No significant difference between blank and DPSC cultures was observed. (**B**) Comparison of glutamate release between DPSC neuron without any drug (control) and those with the 4 different peptides (Arg0, 1, 2, 3). Arg3 demonstrated the greatest anti-wrinkle efficacy with a reduction of 43% in the release of glutamate. (*p < 0.05; **p < 0.01).

Arg0 inhibits the formation of a ternary SNARE complex and in turn inhibits neuronal exocytosis and decreases wrinkle formation. In this study, the degree of glutamate release is used as an estimate for acetylcholine release, as glutamate is the most prevalent excitatory neurotransmitter in the nervous system^28^ and can be detected more easily than acetylcholine which is present in less quantity. Inhibition of glutamate release has also been used previously as a validated cell assay for measuring the potential activity of Arg0 on the inhibition of neuronal exocytosis^29^. Primary human dental pulp stem cells derived neurons (DPSC neurons) were chosen due to its ability to differentiate into functionally active neurons and reduced problems associated with epigenetic changes and re-programming, as compared to induced pluripotent stem cells^30^. Comparing to the undifferentiated dental pulp stem cells (DPSC), a significantly higher fluorometric readout (indicative of the amount of glutamate released) was observed in the neuronally differentiated DPSC neuron group, as compared to blank and DPSC group (Fig. 5A). There was no significant difference between the blank and DPSC group (Fig. 5A). This indicated a successful differentiation of DPSC into functional DPSC neurons with exocytotic functions.

Subsequently, the DPSC neuron cells were treated with the 4 different peptide analogues. A decrease of ~13% in the release of glutamate as compared to control was demonstrated by Arg0. A similar efficacy was demonstrated in Arg1, with a decrease of ~14% in the release of glutamate as compared to control. While Arg2 has good skin permeation as demonstrated above, a relatively small efficacy with a decrease of only ~4% in the release of glutamate as compared to negative control was demonstrated. The highest efficacy for *in vitro* inhibition of the release of glutamate as compared to negative control, was demonstrated by Arg3 (~43%). The greater the inhibition of glutamate release, the higher the efficacy in reducing wrinkles. Therefore, the *in vitro* efficacy is as such, in a descending order of Arg3, Arg1, Arg0, Arg2 (Fig. 5B).

## Discussion

In this study, we demonstrated the use of chemical structure modifications to improve the transdermal delivery of small peptides. Out of the 3 modified peptide analogues, Arg2 and Arg3 had consistently greater cumulative amount of peptides permeated through the skin after 24 hrs, while Arg1 had consistently less cumulative amount of peptide permeated through the skin after 24 hrs, as compared to Arg0. Also in this study, peptide analogues dissolved in PG demonstrated higher amount permeated through the skin.

Stratum corneum, the outermost barrier of skin, is made up of corneocytes and acts as a rate limiting barrier to transdermal drug delivery. This layer of skin is lipophilic and allows only small and moderately lipophilic molecules to permeate passively into the deeper layers of the skin. On the other hand, the stratum corneum restricts the transdermal delivery of peptides and proteins, which have high molecular weights and are hydrophilic or charged in nature^24^. Therefore, when the lipophilicity was increased from Arg0 to Arg1 to Arg2 to Arg3, there was in general, a corresponding higher amount of peptide permeating through the skin by 24 hrs. However, to increase the lipophilicity, there was an accompanying increase in molecular weight. A balance between the two components is required. This was illustrated in the case of Arg1, where it is more lipophilic than Arg0, but yet, had lower skin permeation as compared to Arg0 (most likely due to the increase in molecular weight). Similar findings was observed when we compared Arg3 to Arg2.

PG is an aliphatic alcohol commonly found in cosmetics. Recent data showed that PG has been used in over 9000 cosmetic formulations (out of a total of 34391 formulations reported) and its concentrations ranges from 0.0008% to 99%^31^. For leave-on formulations, the highest PG concentration is 73%^31^. PG has also been used as a co-solvent for poorly soluble materials and/or to enhance drug permeation through skin from topical preparations^26^. Unlike some penetration enhancers, PG does little to the skin as no evidence was found for an effect of PG pre-treatment on intercelluar lipid lamellar structure or for uptake of PG into corneocytes^32^. Its proposed mechanism of action is to partition into the stratum corneum and increase permeant solubility in and thus permeant flux through the stratum corneum^33^. Therefore, the higher the amount of PG, the greater the penetration enhancing effect.

However, this is different from what we expected as shown in Fig. 4. Based on the permeation profiles of the different peptide analogues at varying PG concentrations (Fig. 4), the amount permeated through the skin for each peptide analogue was not always positively correlated to the concentration of PG. In the case of Arg0, 70% PG was demonstrated to give the greatest cumulative amount permeated through the skin and this amount was significantly different (p < 0.05) from that of all other concentrations of PG. For Arg1, Arg2 and Arg3, the PG concentration that achieves the highest amount of peptide analogue permeated through the skin by 24 hrs was 100%. However, the amount permeated through the skin was not significantly different from all concentrations of PG.

One possible explanation for the varying effect of PG on skin permeation is the effect of PG on peptide solubility. In the event of Arg0, at all concentrations of PG from 70% w/w and below, the amount of Arg0 used in the donor compartment are all 1% w/w. Therefore, as the concentration of PG increases, the amount of Arg0 permeated through the skin increases as well since a greater proportion of PG can partition into the stratum corneum, bringing the peptide through to the stratum corneum and beyond. Using neat PG, the saturated solution in the donor compartment was only 0.2% w/w. Due to lower amount of dissolved peptide, regardless of how much PG partition into the stratum corneum, the total amount that permeated through will still be minimal.

From the results of Ostrenga *et al*.^34^, there was a dramatic decrease in permeability of fluocinolone acetonide for PG treated skin versus water based treated skin. This was later confirmed by other groups, that one likely interaction of concentrated PG solutions with skin is dehydration of corneocytes due to the hygroscopic nature of PG. When the skin becomes dehydrated, the permeation of peptide decreases. The interaction of three factors, (1) peptide solubility; (2) partitioning effect of PG; (3) dehydrating effect of concentrated PG could have resulted in the erratic permeation profiles seen in Fig. 4C and D for Arg2 and Arg3.

Structural modification has been successfully used to enhance peptide permeability across intestinal and rectal barriers^35^. However, this method remains largely unexplored for transdermal delivery of peptides and the knowledge gained from this study will be useful. Through the structural modifications, the pKa values of the amino acid side chains were reduced and the side chains were less likely to protonate to form charged ions. Although there is a corresponding increase in molecular weight from the structural modifications, it appeared that the benefits from reducing formation of charged ions and increased logP outweighed the disadvantages associated with increased molecular weight for the purpose of crossing the stratum corneum. Other research groups have attempted chemical structure modification to various peptide compounds to improve transdermal delivery of the peptide. The most common strategy is to synthesise lipophilic derivatives of peptides which can be made either to increase the encapsulation efficiency into liposomes or directly increase their delivery through skin^36^. Foldvari *et al*. demonstrated the use of liposomes and fatty acylation as ways to increase interferon alpha delivery into cutaneous layer for about five to six times greater than the parent protein, for the treatment of genital warts^37,38^. The coupling of a short chain lipoamino acid to a tetrapeptide has also been demonstrated to enhance its delivery across human epidermis, from almost 0% that permeated through the skin for the original peptide, to about 2% that permeated through the skin for the lipophilic derived peptide. A similar study by Namjoshi *et al*. in 2014 also used similar method to enhance a peptide permeation through human epidermis for management of psoriasis^39^. Other structural modifications that have been performed for oral dosing of peptide previously, includes cyclisation^40^ and PEGylation^41^, both of which confer benefits to the systemic stability of the peptide.

In terms of efficacy for potential wrinkle reduction, similar results were demonstrated by Arg1 as compared to Arg0, due to its largely similar chemical structure and physiochemical properties. On the other hand, the highest efficacy was demonstrated by Arg3. This may be attributed to an increased uptake of Arg3, due to a favourable logP of 1.75. The increased uptake was not seen in the case of Arg2. The poor efficacy of Arg2 may be attributed to a reduced cellular uptake of Arg2, due to an increased molecular weight of 1085 Da, while without a significant change in logP, as compared to Arg0. Yet, the relatively higher amount of Arg2 that permeated the skin may still provide an advantage in the use of Arg2 as an alternative to Arg0, alongside Arg3. The results of Arg2 has shown that the permeation through skin can be different from that through cell membranes.

To our best knowledge, this is the first study to conduct multiple modifications on a single peptide compound to enhance transdermal delivery and it has been demonstrated that Arg 2 and Arg 3 were able to permeate through human cadaver skin consistently greater than Arg0 across all compositions of PG. However, molecular modification may affect the peptide’s pharmacological properties and hence must be carefully assessed to ensure that the enhanced delivery analogue has equally good safety and efficacy profiles as compared to the parent compound. Preliminary results from the *in vitro* inhibition of glutamate release study demonstrated the relative efficacy of the various analogues as compared to Arg0. Together with the *in silico* computed safety profile and *in vitro* skin permeation profiles, it appears that Arg2 and Arg3 may be viable alternatives to Arg0 in the topical treatment of wrinkles. However, while the application of molecular modification to enhance transdermal delivery of peptide is relatively new and has merits on a scientific basis, it may be less convenient from a regulatory viewpoint as chemical modification will imply that a new compound is generated. Nonetheless, the main molecular structures of the peptide analogues remain the same (hexapeptide), which may help to acquire regulatory clearance for cosmetic applications.

A candidate drug is expensive to develop (average cost being USD 1 billion per drug) and unlikely to make it to market, with high attrition rates^42^. With pharmacokinetics and pharmacodynamics being one of the major causes of drug development failure in Phase I clinical trials^43^, this study is focused on the skin permeation profiles of the 4 peptide analogues to identify any potential pitfalls in the pre-clinical drug development phase, before any full scale investigation on efficacy of the peptide analogue is conducted in the near future.

## Methods

### Materials

Arg0 and its peptide analogues were synthesised by Kaijie Peptide Company (Sichuan, China) and used as received. Propylene glycol was purchased from Sigma Aldrich (Singapore). All water used were deionised millipore water, employed from a Millipore DirectQ3^®^ system (Massachusetts, USA). 10X phosphate buffered saline (PBS) was purchased from Vivantis Technologies Sdn Bhd (Selangor, Malaysia). Dulbecco’s Modified Eagle’s Medium, supplemented with fetal bovine serum and penicillin/streptomycin were purchased from Invitrogen (California, USA). Neural induction media consisting of Neurobasal®-A medium was purchased from Gibco (Life Technologies, USA) and supplemented with B-27® from Invitrogen (50 × B-27® supplements stock solution, Invitrogen, California, USA), penicillin/streptomycin (Gibco, Life Technologies, USA), EGF (Sigma Aldrich, Merck, Germany) and bFGF (Life Techologies, USA). L-glutamine was purchased from Sigma Aldrich (Merck, Germany). All other reagents used were of analytical grade.

### Synthesis of Peptides

Briefly, the peptide analogues were synthesised through conventional methods for solid phase chemical peptide synthesis using FMOC based synthetic methodology on Rink-amide resin with modifications specific to each of the compounds. Purification of the final product were achieved by preparative HPLC and other common purification methods.

### Physicochemical Properties of Peptide Analogues

*In silico* prediction of physicochemical and toxicity properties of all 4 peptide analogues were performed using Advanced Chemistry Development (ACD) Labs Percepta software (Advanced Chemistry Development Inc., Toronto, Ontario, Canada).

### Preparation of Saturated Peptide Solution

The solubility of the various peptide analogues was determined for water and PG to prepare saturated peptide solution. Excess peptide was added into each solvent inside a 2 mL Eppendorf tube which was kept shaking in an incubation orbital shaker (Certomat S-1, Satorius, Germany) for 48 h. Entire set up was kept at room temperature. The samples were then centrifuged at 10000 rpm for 3 minutes. Subsequently, the supernatant was transferred into an amber coloured ampoule for assay.

### Skin Preparation

Human dermatomed skin was obtained from Science Care (Arizona, USA). The skin tissues were excised from the thighs of Caucasian male cadaver, who died at the age of 43. The use of cadaver human skin for this study has been reviewed by the National University of Singapore Institutional Review Board (IRB) and subsequently exempted because the cadaveric tissues used in this study were without identifiable private information, in accordance to the NUS IRB-GUIDE-021 Guidelines on Research, using cadavers or cadaveric tissue specimens. Integrity of cadaver skin was investigated using visual inspection of the skin before use to ensure no visible pores or breaks in the skin surface. In addition, any compromise in skin integrity can be identified through a rapid and large increase in the amount of permeated peptide through the skin, at the first time point of sampling. The results of replicates with huge amount of peptide permeated through the skin by 1 hr time point will be discarded.

### *In Vitro* Skin Permeation Experiment

Vertical Franz diffusion cells with an effective exposed area of 1 cm^2^ were used. The skin samples were mounted onto the Franz diffusion cells with epidermis facing up. The donor cell contained 400 µL of saturated solution, while the receptor cell contained 4.8 mL of 1X PBS. Compounds with solubility higher than 1% w/w were maintained at 1%w/w in the donor compartment in consideration of the cost of eventual product. The cells were placed inside a chamber with temperature controlled at 32 °C (comparable to the physiological temperature of the skin surface). Magnetic stirrers in the receptor cells stirred at a speed of 180 rpm. All the receptor solutions were withdrawn at pre-set time intervals and replaced with fresh ones. The receptor solutions were subjected to the measurement of peptide permeated by high performance liquid chromatography (HPLC). Each set of experiment were performed in triplicates.

### HPLC for Drug Analysis

The amount of peptide permeated was determined by using Hitachi L2000 LaChrome Elite HPLC system with Agilent Zorbax Extend-C18 column (4.6 mm × 75 mm × 3.5 µm, 80 Å). The mobile phase consisted of mobile phase A (0.1%v/v trifluoroacetic acid in water) and mobile B (0.1%v/v trifluoroacetic acid in acetonitrile) with an isocratic elution program with ratios of solvents A and B as follows for the various peptide: Arg0 (9:1); Arg1 (17:3); Arg3 (39:1); Arg5 (39:1). The flow rate was set at 1 mL/min. The injection volume was 20 µL for each sampling and ultraviolet detection was performed at a wavelength of 215 nm. A calibration curve was conducted using the respective standard solutions from 1 ppm to 1000 ppm.

### DPSC Cell Culture

Primary human dental pulp stem cells (DPSC) from a single donor (DPF003, AllCells, USA) were cultured in a complete growth media containing Dulbecco’s Modified Eagle’s Medium, supplemented with 10% fetal bovine serum and 1% penicillin/streptomycin. Cell cultures were kept at 37 °C in a humid incubator, supplied with 5% carbon dioxide. The cells used were between passage 4 and passage 9 for all experiments.

### Differentiation of DPSC into DPSC Neurons

DPSC was seeded in 24 well plate with complete growth media as discussed above and left uninterrupted for 24 hours for cells to adhere to the well plate, in a humid incubator kept at 37 °C with 5% carbon dioxide throughout the entire duration of study. Subsequently, complete growth media was removed and cells were rinsed with Dulbecco’s phosphate-buffered saline. For all subsequent duration, the culture media was changed to a neural induction media that consists of Neurobasal®-A medium, 1 × B-27® supplements, 1% penicillin/streptomycin, 20 ng/mL EGF and 40 ng/mL bFGF for 12–14 weeks^44^. Three hundred and fifty µL of neural induction media was used per well. The media was changed every three days by completely removing old media and adding fresh media.

### *In Vitro* Inhibition of Glutamate Release

DPSC neurons were used for the glutamate release study. 4 mM of Arg0 with 200 mM of L-glutamine in PBS was prepared and loaded into individual DPSC neuron cell culture with equal volume of culture media to obtain a final concentration of 2 mM Arg 0 and 100 mM of L-glutamine, for a total duration of 5 h. The same was replicated for Arg1, while saturated solution of Arg2, Arg3 in PBS were used due to its poor solubility in water. Saturated solution of Arg2 and Arg3 in PBS were prepared with the method as described above. Final concentration of 1.35 mM and 1.27 mM in culture media was obtained for Arg2 and Arg3 respectively. The results were subsequently normalised to 2 mM of drug loaded. Following drug loading of 5 h, culture media was removed and washed with PBS. Depolarising media was added to the individual DPSC neuron culture to trigger the exocytosis of glutamate. Depolarising media was prepared using 20 mM of HEPES, 6 mM of calcium chloride, 75 mM of potassium chloride and 66 mM of sodium chloride in deionised water. After 23 h of depolarisation, contents of the cell culture were withdrawn and assay reagents were added for subsequent detection via a fluorometric plate reader (Infinite m200, Tecan Trading AG, Switzerland). The content of glutamate was detected via the use of a commercial fluorometric glutamate assay kit, ab138883 (Abcam plc., Cambridge, UK). Briefly, the coupled enzyme system catalysed the reaction between glutamate and NADP+ to produce NADPH, which is specifically recognized by the NADPH sensor and recycled back to NADP+. During the reaction, a red fluorescence product is produced, which in turn was detected in a fluorescence microplate reader at Ex/Em = 540/590 nm.

### Data Analysis

All data were collated and prepared using GraphPad Prism 6 (GraphPad Software Inc, CA, USA) for any graphical outputs. All results were presented as mean ± standard deviation. Statistical analysis was performed by one-way analysis of variance followed by Tukey *post hoc* test using IBM SPSS Statistics 21.0 (IBM, New York, USA). A probability value of p < 0.05 was considered statistically significant.
