# Author Correction: Enhanced Skin Permeation of Anti-wrinkle Peptides via Molecular Modification

**DOI:** 10.1038/s41598-018-24806-0

**Published:** 2018-04-20

**Authors:** Seng Han Lim, Yuanyuan Sun, Thulasi Thiruvallur Madanagopal, Vinicius Rosa, Lifeng Kang

**Affiliations:** 1 Department of Pharmacy, Faculty of Science, National University of Singapore, Singapore, 117543 Singapore; 2 Department of Pharmacy, Tongji Hospital, Tongji Medical College, Huazhong University of Science and Technology, Wuhan, 430030 China; 3 Faculty of Dentistry, National University of Singapore, Singapore, 119083 Singapore; 4 Present Address: Faculty of Pharmacy, University of Sydney, Pharmacy and Bank Building A15, Sydney, NSW 2006 Australia

Correction to: *Scientific Reports* 10.1038/s41598-017-18454-z, published online 25 January 2018

The original version of this Article contained a typographical error in the spelling of the author Thulasi Thiruvallur Madanagopal, which was incorrectly given as Thulasi Madanagopal Thiruvallur. This has now been corrected in the PDF and HTML versions of the Article.

